# Real-world assessment of clinical outcomes of first-line treatment in metastatic papillary renal cell carcinoma

**DOI:** 10.1093/oncolo/oyaf240

**Published:** 2025-08-04

**Authors:** Manon De Vries-Brilland, Zineb Hamilou, Sunita Ghosh, Daniel Y C Heng, Lori A Wood, Naveen S Basappa, Christian K Kollmannsberger, Jeffrey Graham, Bimal Bhindi, Antonio Finelli, Georg A Bjarnason, Dominick Bosse, Frederic Pouliot, Vincent Castonguay, Rodney H Breau, Ramy R Saleh, Eric Winquist, Aly-Khan A Lalani, Denis Soulières

**Affiliations:** Department of Medical Oncology, Integrated Centers of Oncology (ICO) Paul Papin, Angers 49055, France; Centre Hospitalier de l’Université de Montréal, Montreal, QC H3C 3J7, Canada; Centre Hospitalier de l’Université de Montréal, Montreal, QC H3C 3J7, Canada; Department of Oncology, University of Alberta, Edmonton, AB T6G 1Z2, Canada; Tom Baker Cancer Centre, University of Calgary, Calgary, AB T2N 2T9, Canada; Queen Elizabeth II Health Sciences Centre, Dalhousie University, Halifax, NS B3H 2Y9, Canada; Cross Cancer Institute, University of Alberta, Edmonton, AB T6G 1Z2, Canada; British Columbia Cancer Agency, Vancouver Centre, Vancouver, BC V5Z 4E6, Canada; CancerCare Manitoba, University of Manitoba, Winnipeg, MB R3E 0V9, Canada; Southern Alberta Institute of Urology, University of Calgary, Calgary, AB T2V 1P9, Canada; Division of Urology, Department of Surgery, University of Toronto, Toronto, ON M5T 1P5, Canada; Sunnybrook Odette Cancer Centre, University of Toronto, Toronto, ON M4N 3M5, Canada; Division of Medical Oncology, Department of Medicine, University of Ottawa, Ottawa, ON K1H 8L6, Canada; CHU de Quebec, Université Laval, Quebec City, QC G1R 2J6, Canada; Hotel Dieu de Quebec, Quebec, QC G1R 2J6, Canada; Ottawa Hospital Research Institute, University of Ottawa, Ottawa, ON K1H 8L6, Canada; Department of Medical Oncology, McGill University Health Centre, Montreal, QC H4A 3J1, Canada; Division of Medical Oncology, London Health Sciences Centre & Western University, London, ON N6A 5W9, Canada; Department of Medical Oncology, McMaster University, Hamilton, ON L8S 4L8Canada; Centre Hospitalier de l’Université de Montréal, Montreal, QC H3C 3J7, Canada

**Keywords:** metastatic papillary renal-cell cancer, immunotherapy, TKI, first-line systemic treatment, CKCis

## Abstract

**Background:**

Papillary renal cell carcinoma (pRCC) is the most common non-clear cell RCC (nccRCC), representing up to 15% of RCC cases. Phase 2 trials have evaluated first-line (1L) immunotherapy (IO)-based treatment in nccRCC, but with heterogeneous cohorts and limited comparative data. The specific value of IO for metastatic pRCC (mpRCC) remains unquantified.

**Methods:**

We analyzed prospectively collected data from the Canadian Kidney Cancer Information System to assess the efficacy of 1L systemic therapy in mpRCC with IO-based regimens vs tyrosine kinase inhibitors (TKI). The primary endpoint was time-to-treatment failure (TTF). Secondary endpoints included overall survival (OS), objective response rate (ORR), and treatment-related adverse events (TRAEs). Analyses were adjusted (adj) for IMDC risk groups.

**Results:**

From 2011 to 2024, 197 mpRCC patients received 1L therapy: 70 with IO (alone or in combination) and 127 with TKI. Median follow-up was 21.6 months. Median TTF was 9.9 months with IO vs 5.9 months with TKI (adjHR: 0.62 [0.43-0.91], *P* = .01). Median OS was 36.9 months with IO vs 23.2 months with TKI (adjHR: 0.54 [0.3-0.9], *P* = .018). Objective response rate was 37% with IO vs 23% with TKI (adjOR: 2.2 [0.95-5.2], *P* = .07). The TKI-IO subgroup showed the longest TTF (16.9 months, adjHR: 0.47 [0.26-0.85], *P* = .01) and OS (not reached, adjHR: 0.26 [0.08-0.83], *P* = .02), compared to TKI. Grade 3-5 TRAEs occurred in 31% (IO) vs 27% (TKI).

**Conclusions:**

This real-world study highlights the benefit of IO-based treatment in mpRCC, particularly in the TKI-IO subgroup. Our findings may inform further trials evaluating 1L IO in mpRCC.

Implications for practice This study provides real-world evidence supporting the use of immunotherapy, especially in combination with targeted therapies, as an effective first-line treatment for patients with metastatic papillary renal cell carcinoma, a rare and understudied form of kidney cancer. Compared to targeted therapy alone, immunotherapy-based regimens were significantly associated with longer treatment duration and improved survival outcomes. These findings suggest that immunotherapy combinations could become a preferred approach for this patient population. This work also highlights the need for prospective trials focused on papillary kidney cancer to optimize treatment strategies and improve patient care in clinical practice.

## Introduction

The non-clear cell renal cell carcinoma (nccRCC) category encompasses diverse tumors with distinct histological and molecular profiles.^1^ Historically, compared to metastatic clear cell RCC (ccRCC), metastatic nccRCC has exhibited significantly lower response rates, progression-free survival (PFS), and OS.^2^^,^^3^ Therapeutic strategies for metastatic nccRCC have historically mirrored those for ccRCC due to limited dedicated trials.^4–6^ Papillary renal cell carcinoma (pRCC) is the most common subtype of nccRCC,^7^^,^^8^ accounting for up to 15% of RCC cases.^9^ Historically, pRCC was divided into Type 1 and Type 2,^10^ but the 2022 World Health Organization classification eliminated these subtypes due to evidence suggesting a single origin and progression from low- to high-grade forms,^9^^,^^11^ alongside frequent mixed phenotypes and varied molecular underpinnings.^12^ The PAPMET phase II trial specifically enrolled mpRCC patients and established cabozantinib as the preferred targeted therapy, demonstrating significantly superior PFS and higher response rates compared to sunitinib.^13^ Although no statistically significant OS benefit was observed, the trial was not powered for OS, and these findings underscore the need for further prospective evaluation in this rare population.^14^

The clinical efficacy of immunotherapy (IO) treatment is established in metastatic ccRCC, as monotherapy^15^ or in combination with IO^16^ or targeted therapy.^17–20^ However, these pivotal studies evaluating immune checkpoint inhibitors (CPIs) excluded nccRCC. Small retrospective cohorts report variable CPI efficacy, as a single agent, in metastatic pRCC (mpRCC), with objective response rates (ORRs) ranging from 8% to 25%.^21–26^ A series of phase II trials have guided treatment selection for patients with nccRCC.^27^ Notably, first-line (1L) phase II trials have evaluated IO in combination with IO or tyrosine kinase inhibitors (TKIs) in nccRCC, with ORR ranging from 28.8% to 54%,^28–30^ but these cohorts remain heterogeneous. Although several single-arm trials suggest clinical benefit of IO-based regimens, their efficacy has not been compared with TKI monotherapy in randomized studies, specifically in mpRCC. Importantly, there is no universal standard of care for mpRCC, as different approaches are recommended across international society guidelines.^31^^,^^32^ The optimal treatment strategy remains undefined, and further information must be gained on optimal combinations.^33^

Our objective was to evaluate the efficacy of first-line (1L) systemic therapy using either IO-based or VEGFR-TKI treatments specifically for patients with mpRCC.

## Methods

Data were collected from the Canadian Kidney Cancer Information System (CKCis), a curated prospective national cohort comprising patients with kidney cancer from 15 academic centers across Canada. The CKCis database has been validated as representative of the national kidney cancer population.^34^ Ethics approval to collect deidentified patient information was obtained at all participating centers.

Eligible patients had a confirmed diagnosis of mpRCC, initiated 1L treatment between January 2011 and January 2024. Baseline characteristics, treatment outcomes, and safety data were collected.

The primary endpoint was time-to-treatment failure (TTF), defined as the time from 1L therapy initiation to the date of discontinuation of the treatment or death, or date of discontinuation for toxicity or censored at the last follow-up date.

Secondary endpoints included overall survival (OS), ORR, and treatment-related adverse events (TRAEs) necessitating dose or schedule modifications. Overall survival was measured from treatment initiation to death from any cause or censoring. Objective response rate, defined as the proportion of patients achieving complete (CR) or partial response (PR), was assessed per RECIST 1.1 criteria.^35^

Descriptive statistics summarized baseline clinical variables by treatment group. Continuous variables were reported as means (± SD) for normal distributions or medians (interquartile range) for non-normal distributions. Kaplan-Meier estimates with 95% CI were calculated for OS and TTF with log-rank tests comparing survival curves. Cox proportional hazards models, adjusted for International Metastatic Renal Cell Carcinoma Database Consortium (IMDC) risk categories,^36^ were used to calculate adjusted hazard ratios (adjHRs) for OS and TTF as well as adjusted odds ratios (adjORs) for ORR, with corresponding 95% CI. Sensitivity analyses were conducted for IO-IO (nivolumab-Ipilimumab) vs TKI monotherapy and IO-TKI vs TKI treatments. Subgroup analyses by sarcomatoid component were also described.

All statistical tests were 2-sided, with significance set at *P* < .05. Analyses were performed using SAS version 9.3 (SAS Institute, Inc.).

## Results

Between 01/2011 and 01/2024, a total of 197 patients with mpRCC pts were included in this analysis. Within this cohort, 70 received 1L IO-based therapy (36%), and 127 received TKI monotherapy (64%). Of those who received IO-based therapy, 14% were treated with an IO-IO combination (*n* = 27), 15.5% with an IO-TKI combination (*n* = 30), and 6.5% with IO single agent (*n* = 13). The most common agent used in the TKI group was sunitinib (45%), and only 6.5% received cabozantinib (Table 1). Among the entire cohort, 104 patients (53%) received second-line therapy: 27 patients from the IO-based group, all of whom were treated with a TKI, and 77 patients from the TKI monotherapy group, of whom 21 (20%) received an IO in the second line.

**Table 1.** Baseline characteristics and type of treatment.

	Overall cohort	IO-based group	TKI monotherapy group	*P*-value
	*n* = 197	*n* = 70 (36%)	*n* = 127 (64%)	
**Median age in years (range)**	68 (30-89)	69	67	
**Sex male**	154 (78%)	51 (73%)	103 (81%)	.18
**Karnofsky ≥ 80%**	149 (76%)	56 (80%)	93 (73%)	.60
**Baseline IMDC score**				.14
**Favorable**	31 (21%)	15 (29%)	16 (17%)	
**Intermediate**	87 (59%)	30 (58%)	57 (60%)	
**Poor**	29 (20%)	7 (13%)	22 (23%)	
**Unknown**	50	18	32	
**Prior nephrectomy**	158 (80%)	56 (80%)	102 (80%)	.96
**Sarcomatoid component**	12 (8%)	5 (9%)	7 (7%)	.78
**Location of metastases (more than 1 possible)**				
**Lung**	98 (50%)	32 (46%)	66 (52%)	.40
**Liver**	43 (22%)	13 (19%)	30 (24%)	.41
**Bone**	50 (25%)	17 (24%)	33 (26%)	.79
**Lymph nodes**	127 (64%)	40 (57%)	87 (68%)	.11
**Contralateral kidney**	9 (4.5%)	2 (3%)	7 (5%)	.39
**Adrenal**	33 (17%)	12 (17%)	21 (17%)	.92
**Brain**	10 (5%)	3 (4%)	7 (6%)	.71
**Pancreas**	3 (1.5%)	1 (1%)	2 (2%)	.94
**Soft tissue**	47 (24%)	20 (28%)	27 (21%)	.25
**Others**	41 (21%)	15 (21%)	26 (21%)	.87
**Type of therapy**				
**Nivolumab + ipilimumab**		27 (38%)		
**Pembrolizumab + axitinib**		23 (33%)		
**Pembrolizumab + lenvatinib**		7 (10%)		
**Pembrolizumab**		11 (16%)		
**Nivolumab**		2 (3%)		
**Sunitinib**			88 (69%)	
**Pazopanib**			20 (16%)	
**Cabozantinib**			8 (6.5%)	
**Crizotinib**			3 (2.5%)	
**Savolitinib**			7 (6%)	

Abbreviations: IMDC, International Metastatic Renal Cell Carcinoma Database Consortium; IO, immunotherapy; TKI, tyrosine kinase inhibitors.

Most patients were men (78%) and had a Karnofsky performance status greater than or equal to 80% (76%). At the start of 1L treatment, the median age was 68 years (range 30-89). Breakdown by IMDC risk group^37^ was 21% favorable, 59% intermediate, and 20% poor. The majority of patients (80%) had undergone a prior nephrectomy. A sarcomatoid component was observed in 12 patients (8%). Main sites of metastases included lymph nodes (64%), lung (50%), liver (22%), and bone (25%). There were no significant differences in baseline characteristics between 2 groups, as described in Table 1.

The median follow-up time for the entire cohort, calculated from the start of 1L therapy to the date of death or last known follow-up (ie, based on OS), was 21.6 months (mo) (range: 1.6-146.1). The median follow-up in the IO-based group was 20.8 mo (1.6-79.4), and in the TKI monotherapy group, it was 22.7 mo (1.6-146.1).

**Figure 1.**
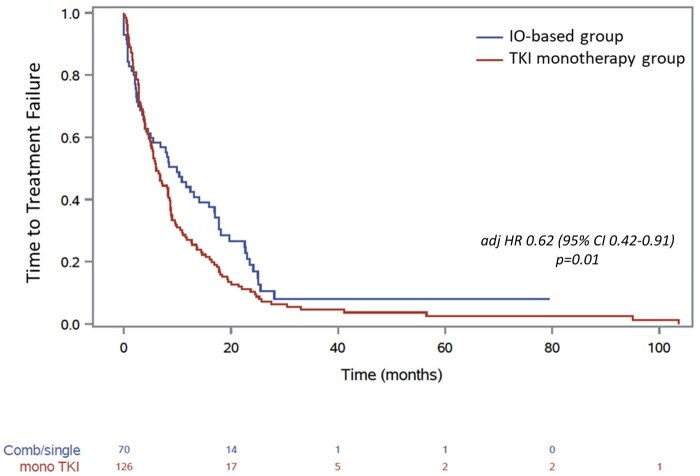
Kaplan-Meier curves for time-to-treatment failure for patients with metastatic papillary renal cell carcinoma receiving first-line immuno-oncology (IO)-based combinations or tyrosine kinase inhibitors (TKIs).

The median TTF with IO-based was 9.9 months (95% CI, 4.5-15.9) vs 5.9 months with TKI monotherapy (95% CI, 4.8-8.2) (Figure 1). After adjusting for IMDC risk group, IO vs TKI monotherapy-based regimens were associated with prolonged TTF (adjHR: 0.62 [95% CI, 0.42-0.91], *P* = .01).

**Figure 2.**
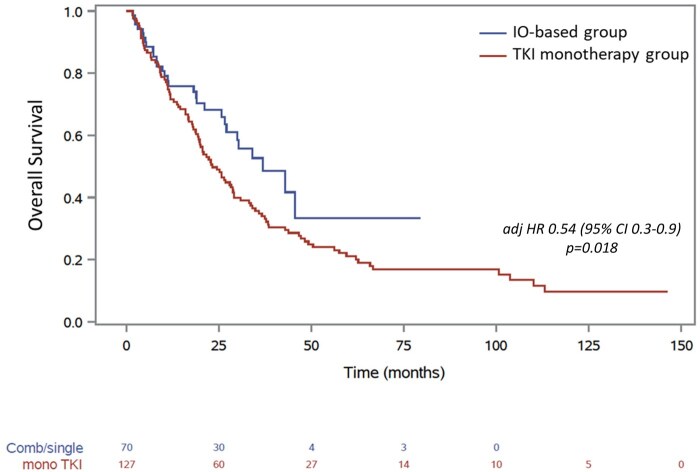
Kaplan-Meier curves for overall survival for patients with metastatic papillary renal cell carcinoma receiving first-line immuno-oncology (IO)-based combinations or tyrosine kinase inhibitors (TKIs).

The median OS with IO-based was 36.9 months (95%CI 26.5, not reached [NR]) vs 23.2 months with TKI monotherapy (95% CI, 19.5-29) (Figure 2). After adjusting for IMDC risk group, IO vs TKI monotherapy-based regimens were associated with prolonged OS (adjHR: 0.54 [95% CI, 0.3-0.9], *P* = .018).

A total of 162 patients were considered evaluable for treatment response. Objective rate response was 37% (95% CI, 25-50) with IO-based, including 2 CR, vs 23% (95% CI, 16-32) with TKI, including 1 CR, as shown in Table 2. After adjusting for IMDC risk group, the OR for ORR comparing IO-based therapy to TKI monotherapy did not reach significance (adjOR: 2.2 [95% CI, 0.95-5.2], *P* = .07) (Table 2).

**Table 2.** Response by type of first-line treatment.

Best response	IO-based group	IO-IO subgroup	IO-TKI subgroup	TKI monotherapy group	AdjOR^a^ (95% CI; *P*-value)
	*n* = 70	*n* = 27	*n* = 30	*n* = 127	
**ORR**	20 (37%)	9 (39%)	9 (38%)	25 (23%)	2.2 (0.95-5.2; *P* = .07)
**CR**	2 (4%)	2 (9%)	-	1 (1%)	
**PR**	18 (33%)	7 (30%)	9 (38%)	24 (22%)	
**SD**	17 (31%)	7 (30%)	8 (33%)	36 (33%)	
**PD**	17 (31%)	7 (30%)	7 (29%)	47 (44%)	
**Unknown**	16	4	6	19	

a Adjusted for IMDC, OR for comparison of ORR between IO-based group vs TKI monotherapy group.

Abbreviations: AdjOR, adjusted odds ratios; CI, confidence interval; CR, complete response; IMDC, International Metastatic Renal Cell Carcinoma Database Consortium; IO, immunotherapy; ORR, objective rate response; PR, partial response; PD, progressive; SD, stable disease; TKI, tyrosine kinase inhibitors.

**Figure 3.**
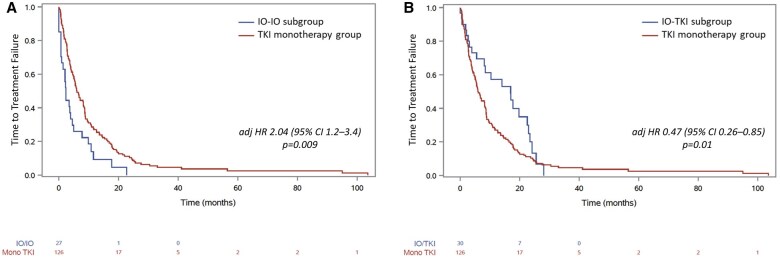
Kaplan-Meier curves for time-to-treatment failure for patients with metastatic papillary renal cell carcinoma receiving first-line IO-IO combination or tyrosine kinase inhibitors (TKIs) (A) and receiving first-line IO-TKI combination or TKI (B). IO, immuno-oncology.

**Figure 4.**
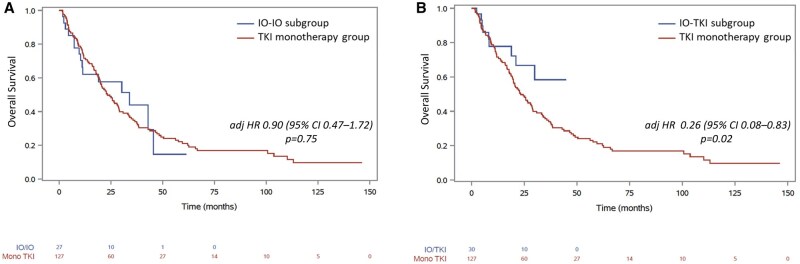
Kaplan-Meier curves for overall survival for patients with metastatic papillary renal cell carcinoma receiving first-line IO-IO combination or tyrosine kinase inhibitors (TKI) (A) and receiving first-line IO-TKI combination or TKI (B). IO, immuno-oncology.

Sensitivity analysis was performed by the type of 1L IO therapy received. Compared to patients treated with TKI monotherapy, those in the IO-IO subgroup had a shorter median TTF of 2.4 months vs 5.9 months, with an adjHR of 2.04 [95% CI, 1.2-3.4] and *P* = .009. No significant difference in OS was observed between the IO-IO and TKI groups, with median OS of 34 months vs 23.2 months, respectively (adjHR: 0.90 [95% CI, 0.47-1.72], *P* = .75) (Figures 3A and 4A, respectively). Among the 155 evaluable patients, ORR on IO-IO was 39% (95% CI, 22-59) with 2 CR and 7 PR and not significantly different compared to TKI-treated patients (adjOR: 1.7 [95% CI, 0.52-5.5], *P* = .38) (Table 2). In contrast, patients in the TKI-IO subgroup had significantly improved outcomes compared to TKI monotherapy, with a median TTF of 16.9 months vs 5.9 months (adjHR: 0.47 [95% CI, 0.26-0.85], *P* = .01), and a longer OS, which was NR vs 23.2 months in the TKI group (adjHR: 0.26 [95% CI, 0.08-0.83], *P* = .02) (Figures 3B and 4B, respectively). Among the 155 evaluable patients, the ORR was 37.5% (95% CI, 21-57), including 9 PR and was significantly higher than the ORR of 23% for TKI monotherapy (adjOR: 4.2 [95% CI, 1.25-14.3], *P* = .02) (Table 2).

Amongst the 12 patients with sarcomatoid component, the majority of IO-based therapy patients were treated by IO-IO (4/5), and 7 treated by TKI monotherapy. Given the very small sample size, these findings were descriptive, and no statistical comparisons were performed. The median TTF was 2.3 months in IO-based group vs 8.5 months in TKI monotherapy group. The median OS was 34 months in IO-based group vs 19 months in TKI group. In the IO-based group, 1 CR and 2 PR were reported in the 4 patients treated with IO-IO, and 1 progression (PD) for TKI-IO. In the TKI monotherapy group, there were no responses, but 4 stable disease and 2 with PD in patients treated with a TKI.

In addition, 1L treatment was discontinued due to disease progression in 87 patients. Treatment discontinuation due to toxicity occurred in 51 patients, 34 (29.8%) in the TKI arm and 17 (34.7%) in the IO-based arm. Grade 3-5 TRAEs were reported in 30.8% of IO-treated patients and 27.4% of TKI-treated patients, with no statistical difference (*P* = .51). 27.7% of grade 3-5 toxicities led to discontinuation of TKI monotherapy, and 28.4% of grade 3-5 toxicities led to discontinuation of IO-based therapy (*P* = .9). Three patients experienced grade 5 toxicity, 2 in the IO arm (both myocarditis) and 1 in the TKI arm (dyspnea).

## Discussion

This is the first multicenter study with prospectively collected comparative data providing real-world insights into 1L treatment for mpRCC, contributing to improve understanding of these rare tumors, which are often underrepresented in randomized trials.

After adjusting for IMDC risk groups, we observed a significant associated improvement in TTF and OS, along with a higher ORR in the IO-based group compared to patients receiving TKI monotherapy. These findings are consistent with the established value of IO in 1L mpRCC treatment, aligning with previous prospective^30^^,^^38–40^ and retrospective studies.^41–44^ Similarly, the ARON-1 study, which included 200 mpRCC patients (73 treated with IO-based combinations and 127 with TKI monotherapy), demonstrated an ORR of 41%, median PFS of 17.4 months, and median OS of 28.8 months in favor of IO-treated patients.^42^ Although no conclusion can be drawn from PFS and TTF comparison, these results are consistent with our findings, except for the longer PFS, which may be due to the inclusion of IO monotherapy in our study. A retrospective study by Graham et al., which included 1145 metastatic nccRCC patients (54.9% with pRCC) from the international metastatic RCC database consortium (IMDC), reported a median OS of 28.6 months in the IO group, 16.4 months in the TKI group, and 12.2 months in the mTOR inhibitor group, further supporting improved survival with IO-based therapies. Of note, the Graham study also included patients treated with IO monotherapy, and the median TTF in the IO group was 6.9 months vs 5.0 months in the TKI group and 3.9 months in the mTOR group.^41^ Additionally, the prospective KEYNOTE-427 cohort B study demonstrated promising antitumor activity with 1L pembrolizumab monotherapy in metastatic nccRCC, reporting an ORR of 28.8%, median PFS of 5.5 months, and median OS of 31.5 months in 118 mpRCC patients.^27^ These results suggest that IO-based combination treatment could be preferable to monotherapy in mpRCC.

The optimal IO-based combination remains an open question.^33^ Sensitivity analysis showed no significant OS difference between IO-IO and TKI groups, although TTF appeared lower in the IO-IO group. The phase 3 b/4 CHECKMATE-920 trial, which evaluated nivolumab plus ipilimumab in 18 untreated pRCC patients, reported an ORR of 19.6%, median OS of 21.2 months, and median PFS of 3.7 months, consistent with our results. Recently, the first prospective randomized trial, SUNNIFORECAST, compared dual checkpoint inhibitor therapy to standard of care in 309 patients with nccRCC (57.6% with pRCC). In the overall population, ipilimumab/nivolumab significantly improved 12-month OS rates compared to TKI monotherapy (86.9% vs 76.8%, *P* = .014), although no significant difference in PFS was observed. In the pRCC subgroup, the ORR with ipilimumab/nivolumab was 29%, and the median OS was 28.4 months (95% CI, 18.4-40.9) vs 18.9 months (95% CI, 14.4-32.8) with standard of care, with an HR of 0.84 [95% CI, 0.59-1.21] and 12-month OS rates of 74.6% vs 64.1%, respectively. However, these results did not reach statistical significance.^29^ This cohort remains heterogenous mainly because it comprises different histological subtypes.

The TKI-IO combination demonstrated the best results numerically, with significantly improved TTF and OS compared to TKI monotherapy in our study. Several trials have evaluated IO-TKI combinations in nccRCC, yielding mostly concordant results. The phase 1 b COSMIC-021 trial reported an ORR of 31% with cabozantinib/atezolizumab in nccRCC.^38^ The KEYNOTE-B61 trial, which enrolled 158 advanced nccRCC patients (59% with mpRCC) treated with lenvatinib/pembrolizumab, showed a median PFS of 18 months and median OS not reached. In patients with mpRCC, the ORR was 54%% (95%CI, 38.5%-67.1%).^30^ Similarly, a trial of cabozantinib/nivolumab in 40 patients with pRCC (80%), unclassified RCC (15%), and tRCC (5%) reported an ORR of 48%, a median PFS of 13 months, and a median OS of 28 months.^28^ Ongoing trials, such as CANI (NCT04413123), PAPMET 2 (NCT05411081), PAXIPEM (NCT05096390), and SAMETA (NCT05043090), continue to explore IO-TKI combinations in variant histology RCC.

For the TKI monotherapy group, our results were also consistent with the data in the literature.^14^^,^^45^^,^^46^ For example, our most commonly used agent in the TKI group being sunitinib, the SUPAP trial of sunitinib reported median OS of 17.8 months for type I and 12.4 months for type II pRCC, and corresponding median PFS of 6.6 months and 5.5 months.^45^

This study has limitations inherent to real-world data analyses of efficacy endpoints, despite prospective data collection. We chose TTF as the primary endpoint because it better reflects real-world treatment decisions in a retrospective, multicenter setting. Unlike PFS, which requires standardized imaging not available across centers, TTF captures key clinical events such as discontinuation due to progression, toxicity, or other reasons. We acknowledge its limitations, especially with IO regimens, but it remained the most feasible and consistent measure in our study. There was no central radiological or histological review, particularly relevant given recent changes in pRCC classification.^1^ Although there was no significant difference between the groups, there was a numerical difference in the IMDC risk groups between the IO-based and TKI monotherapy groups, with more patients with a favorable IMDC score and fewer with a poor IMDC score in the IO-based group (29% vs 17% and 13% vs 23%, respectively). Although the findings appear to be driven by the IO-TKI subgroup, the study power limited the ability to compare IO-TKI vs. IO-IO. Another limitation of our study is that, given our study included patients from 2011 onward and the evolving literature, few patients in the TKI group received cabozantinib, which has now been demonstrated to be a preferred TKI.^13^ However, despite this weakness, the randomized ccRCC trials that led to the approval of IO/IO and IO/TKI regimens used sunitinib as the control arm.^16–20^ The only comparative trial to date in nccRCC evaluating IO-IO vs TKI also included a majority of sunitinib-treated patients in the control arm (78% vs 7% cabozantinib-treated).^29^ Finally, the efficacy of IO-containing regimens vs cabozantinib remains unknown. Additionally, only 8% of patients in our study had sarcomatoid features, which is consistent with the literature. However, this low proportion limits the ability to perform robust statistical analyses, and the observed outcomes with IO-IO combinations in this subgroup should be considered hypothesis-generating. We observed an interesting ORR in IO-IO–treated patients, suggesting that this combination may be preferable for sarcomatoid pRCC, as previously demonstrated in sarcomatoid RCC,^47^^,^^48^ even though formal demonstration of correlation with PFS and OS is undocumented in this study. Finally, the CKCis database currently lacks immunohistochemical and transcriptomic biomarker data. According to SUNNIFORECAST's exploratory analysis, the PD-L1 CPS score could be a predictive marker of response to IO-IO combination therapy.^29^ However, this marker remains heterogeneous in pRCC.^49^^,^^50^ It seems essential to be able to identify biomarkers to help personalize the management of these rare tumours.^51^^,^^52^

In summary, this study provides the first real-world comparative data from prospectively collected data on 1L treatment for mpRCC, demonstrating improved TTF and OS with IO-based therapy, particularly in the TKI-IO subgroup, with known and acceptable toxicity. Immunotherapy plays a key role in pRCC management, emphasizing the need for further prospective trials to refine treatment strategies and identify predictive biomarkers for patient selection.

## Data Availability

Our clinical data will be made available on request, subject to justification of the project. For obtaining the data, please contact our corresponding author, Doctor Manon DE VRIES BRILLAND.
